# Age alters the oncogenic trajectory toward luminal mammary tumors that activate unfolded proteins responses

**DOI:** 10.1111/acel.13665

**Published:** 2022-09-15

**Authors:** Edmund Charles Jenkins, Mrittika Chattopadhyay, Maria Gomez, Denis Torre, Avi Ma'ayan, Miguel Torres‐Martin, Daniela Sia, Doris Germain

**Affiliations:** ^1^ Department of Medicine, Division of Hematology/Oncology, Tisch Cancer Institute Icahn School of Medicine at Mount Sinai New York New York USA; ^2^ Rutgers Cancer Institute of New Jersey New Brunswick New Jersey USA; ^3^ Department of Pharmacological Sciences, Mount Sinai Center for Bioinformatics Icahn School of Medicine at Mount Sinai New York New York USA; ^4^ Clinical Genomics Research Group Germans Trias I Pujol Research Institute (IGTP) Barcelona Spain; ^5^ Department of Medicine, Division of Liver Diseases, Tisch Cancer Institute Icahn School of Medicine at Mount Sinai New York New York USA

**Keywords:** aged mammary gland, aging, endoplasmic reticulum, ER stress, estrogen receptor‐alpha, mitochondrial UPR, unfolded protein response, XBP‐1

## Abstract

A major limitation in the use of mouse models in breast cancer research is that most mice develop estrogen receptor‐alpha (ERα)‐negative mammary tumors, while in humans, the majority of breast cancers are ERα‐positive. Therefore, developing mouse models that best mimic the disease in humans is of fundamental need. Here, using an inducible MMTV‐rtTA/TetO‐NeuNT mouse model, we show that despite being driven by the same oncogene, mammary tumors in young mice are ERα‐negative, while they are ERα‐positive in aged mice. To further elucidate the mechanisms for this observation, we performed RNAseq analysis and identified genes that are uniquely expressed in aged female‐derived mammary tumors. We found these genes to be involved in the activation of the ERα axis of the mitochondrial UPR and the ERα‐mediated regulation of XBP‐1s, a gene involved in the endoplasmic reticulum UPR. Collectively, our results indicate that aging alters the oncogenic trajectory towards the ERα‐positive subtype of breast cancers, and that mammary tumors in aged mice are characterized by the upregulation of multiple UPR stress responses regulated by the ERα.

## Graphical abstract

Older women tend to develop estrogen receptor‐alpha (ER󠆴α)‐positive breast cancers, despite having lower levels of estrogen, but this fundamental observation has never been recapitulated in mice, where most studies are performed in young mice. We show that the expression of the same oncogene in the mammary glands of young and aged mice leads to ER󠆴α‐negative and ER󠆴α‐positive, respectively. Tumors in aged mice are characterized by the activation of multiple unfolded protein responses (UPR) driven by the ER󠆴α that is not found in ER󠆴α‐positive cells from young mice. These findings suggest that age dictates not only breast cancer subtypes but also ER󠆴α transcriptional programs.

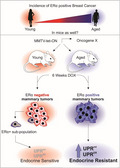

## 1 INTRODUCTION

Breast cancer, like many other cancer types, is an age‐related disease. Breast cancer risk peaks between the ages of 55 and 70, and since the average age at menopause is 51, the majority of breast cancers occur in postmenopausal women. Menopause marks the cessation of ovarian function, the main source of estrogen in women. Yet, the incidence of estrogen receptor‐alpha (ERα)‐positive breast cancers increases rather than decreases with age (Acheampong et al., 2020). While non‐ovarian sources of estrogen exist through the activity of the aromatase enzyme, estrogen levels remain lower in post‐menopausal women relative to pre‐menopausal women. Therefore, the observation of an increase in ERα‐positive breast cancer in older women is counterintuitive and suggests that other selection pressures, independent of estrogen levels, must take place to favor the transformation of luminal ERα‐positive cells relative to ERα‐negative cells with age.

In contrast to what is observed in humans, in mice, the vast majority of genetically engineered models lead to the formation of ERα‐negative mammary tumors. This apparent inability of mouse models to mimic the disease in humans represents a significant limitation for breast cancer research. However, as in most diseases, mammary tumors are studied in young mice almost exclusively with the exception of studies using established human breast cancer cell lines in young and old mice (Gravekamp et al., 2004; Grizzle et al., 2007; Rockwell, 1989).

**FIGURE 1:**
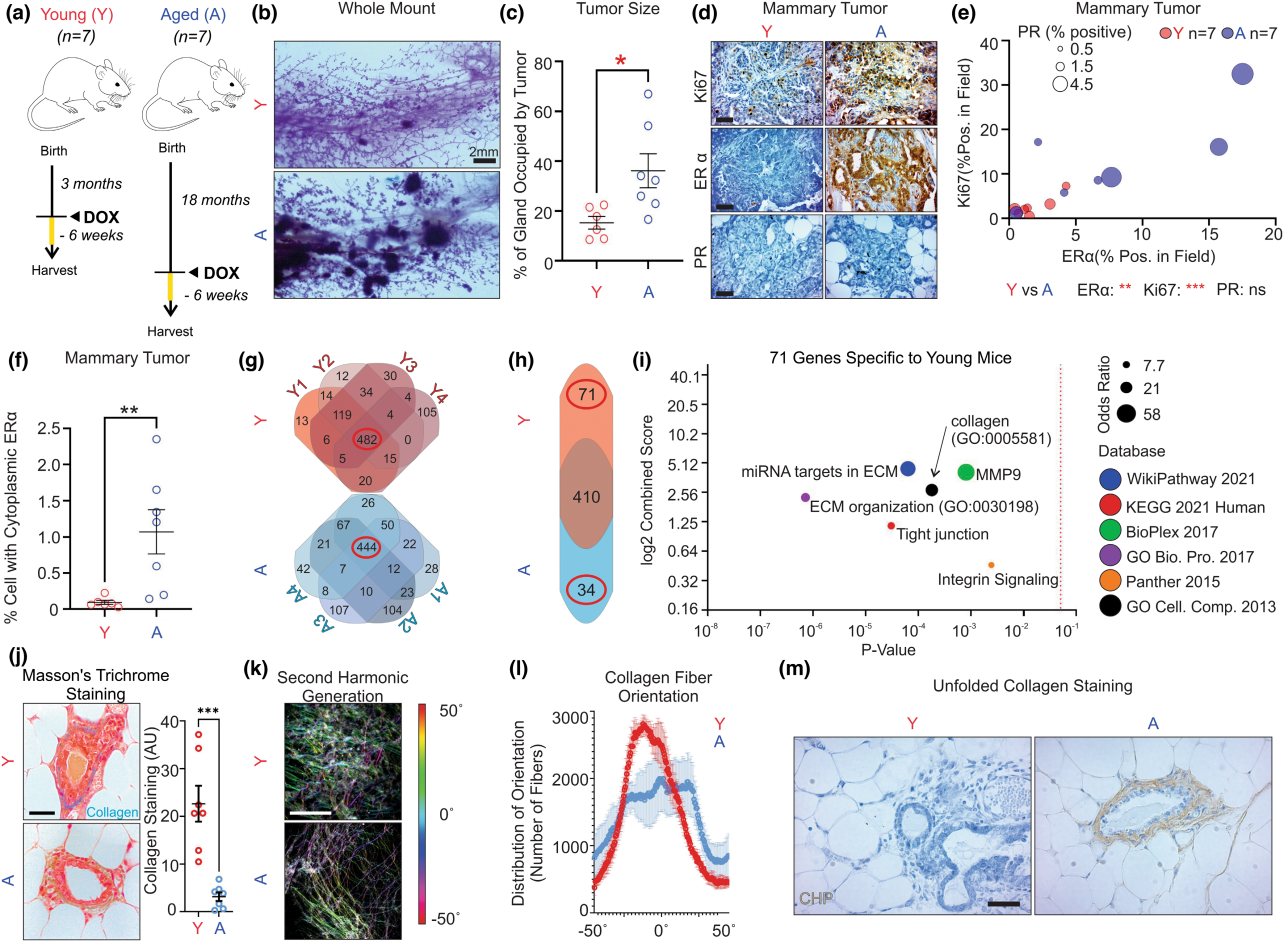
Aged mice develop ERα‐positive mammary tumors and differ in gene expression relative to young mice mammary tumors driven by the same oncogene. (a) Diagram of the experimental plan, in each group *n* = 7 female mice. (b) Representative whole mount of a mammary gland from young and aged mice following treatment with doxycycline to induce tumor formation. (c) Quantification of percentage of mammary gland occupied by tumors in all 7 mice per group. (d) Representative image of IHC of the ERα, PR, and Ki67 in young and aged female‐derived mammary tumors. (e) Quantification of ERα, PR, and Ki67 staining in all mice, where each dot represents one mouse. 5–10 fields were analyzed per mouse. ERα intensity is indicated on the x axis, Ki67 intensity is indicated on the y axis, and the sizes of the dots indicate the intensity of PR staining. Red indicates young; blue indicates aged. (f) Quantification of percentage cells showing cytoplasmic ERα staining in young and aged female‐derived tumors. Significance was determined by the Student's *t*‐test. * *p* < 0.05, ** *p* < 0.005. (g) Graphical representation of number of genes that are either unique or common between young mice (Y1‐4) or aged mice (A1‐4). (h) Venn diagram of genes that are common between group (410), unique to young female (71) or unique to aged female‐derived tumors (34). (i) Graph of p‐value versus log score of indicated pathways. Increasing circle size correlates with odd ratios. (j) Representative Masson's trichrome staining of collagen in the mammary glands of young and aged derived mice (left). Blue indicates collagen staining. Graph of quantification of collagen in all mice is shown (right). (k) Second harmonic imaging (SHG) of representative young female‐derived and aged female‐derived decellularized mammary glands. Colors indicate collagen orientation. (l) Graph of the distribution of the orientation of collagen fibers in young and aged female‐derived decellularized mammary glands. (m) Staining of denatured collagen‐binding peptide in young and aged female‐derived mammary glands. Scale bars indicate 2 mm (panel B) or 100 μm (panels d, j, k, and m).

Therefore, we reasoned that it remains formally possible that activation of an oncogene in older mice may, as in humans, favor the transformation of luminal ERα‐positive cells and the formation of ERα‐positive mammary tumors. We therefore took advantage of the inducible MMTV‐rtTA/TetO‐NeuNT mouse model to induce tumor formation in 3‐month‐old and 18‐month‐old mice. We selected 18 months with the intent to mimic the age at which breast cancer incidence peaks in women (age 55 and 70). Doxycycline was added to the drinking water of mice for 6 weeks in both groups and the mammary glands were harvested for tumor analysis (Figure 1a). We found that aging increases tumor incidence and size (Figure 1b, c). Most importantly, when stained for the ERα by IHC, we found that while very faint staining was observed in the young female‐derived mammary tumors, a drastic increase in ERα staining was observed in mammary tumors derived from older mice (Figure 1d–e). This increase in ERα expression was associated with an increase in the expression of its transcriptional target, the progesterone receptor (PR), and increased proliferation as indicated by staining for Ki67 (Figure 1d–e). Cytoplasmic staining of the ERα was also observed in 1.2% of cells (Figure 1f), which correlates with what has been reported in human breast cancers (Welsh et al., 2012).

To further characterize the differences between the two groups, we performed RNA sequencing of the tumors from the young and aged mice. In young mice, 482 genes were found to be common in all four mice analyzed, while in the aged mice, 444 genes were common in all four mice analyzed (Figure 1g). We then identified genes that are uniquely differentially expressed between the groups. We found that 410 genes were shared between the two groups, 71 were unique to the young group, and 34 were unique to the aged group (Figure 1h, Table S1). Pathway analysis revealed a strong bias toward extracellular matrix and collagen‐related pathways in young mice (Figure 1i). We therefore analyzed collagen using multiple approaches. First, we performed Masson's trichrome staining of collagen and found lower staining in aged female‐derived mammary glands (Figure 1j). Second, we used decellularized mammary glands from both young and aged women and performed a second harmonic generation (SHG) analysis to determine the amount and the orientation of collagen. We found that in agreement with Masson's trichrome staining (Figure 1j), the amount of collagen signal is lower in mammary glands derived from older mice. (Figure 1k). Further, when fibers were color‐coded based on their orientation, we found a wider range of different orientations in the aged mammary glands (Figure 1l). Third, we performed staining using a peptide that selectively binds to denatured collagen and found that this peptide binds strongly to ducts of aged mammary glands but not young mammary glands (Figure 1m). These results are in strong agreement with the recent proteomic analysis of young and aged mammary glands, which revealed a significant decrease and alteration in the collagen during aging (Bahcecioglu et al., 2021). Therefore, we view these findings as an internal validation of our RNAseq analysis.

**FIGURE 2:**
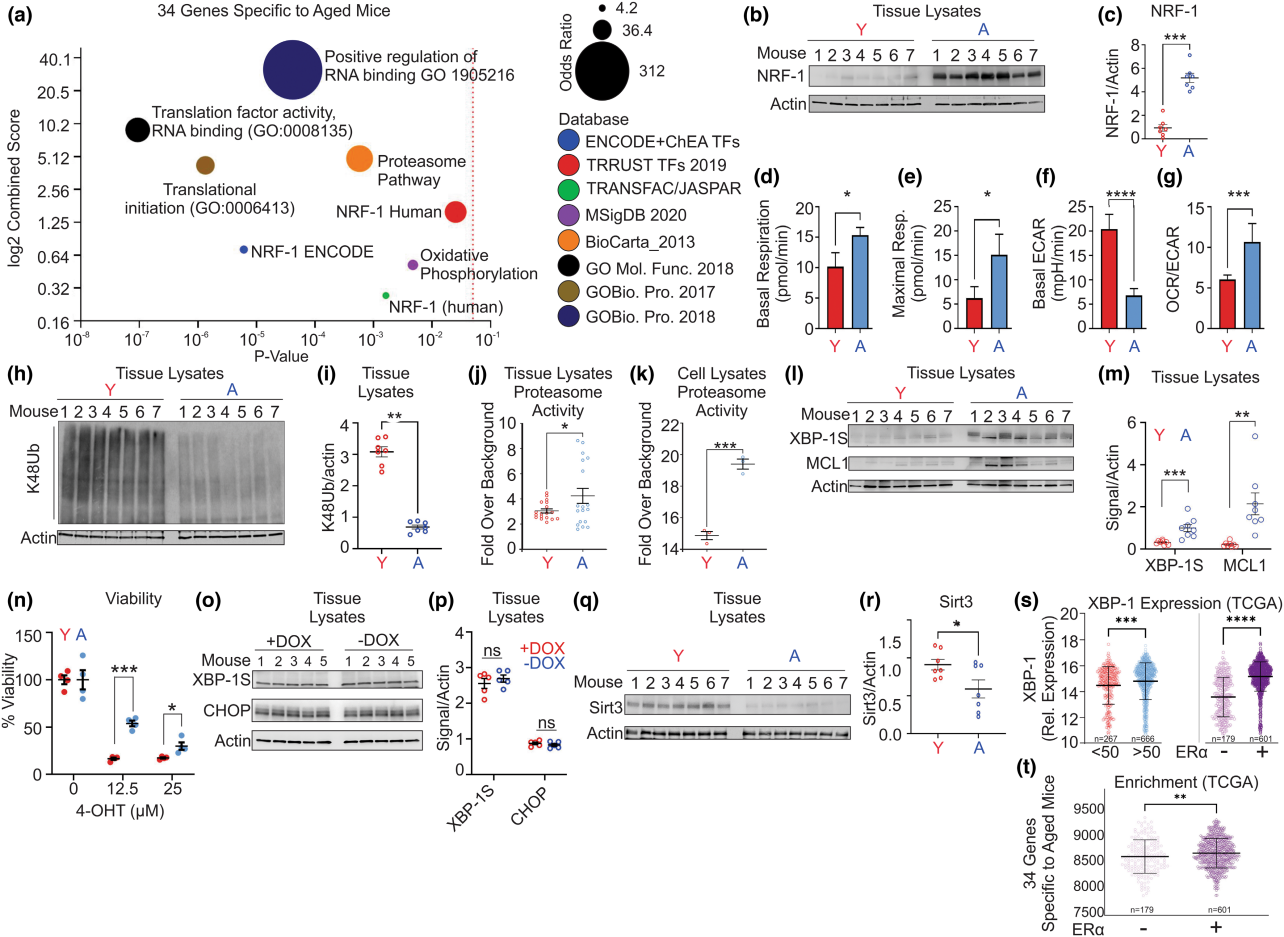
Aged female‐derived mammary tumors activate the ERα axis of the UPR^mt^, the XBP‐1 axis of the UPR^ER^, and anti‐apoptotic MCL‐1. (a) Graph of *p*‐value versus log score of indicated pathways. Increasing circle size correlates with odd ratios. Red dotted line indicates *p* = 0.05. (b) Western blot of NRF‐1 in young and aged female‐derived mammary tumors. Actin was used as a loading control. (c) Quantification of the Western in B. (d) Basal and (e) maximal respiration in young and aged female‐derived mammary tumor cell lines. (f) Baseline extracellular acidification rate (ECAR) in young and aged female‐derived mammary tumor cell lines. (g) Baseline ratio of oxygen consumption rate (OCR) over extracellular acidification rate (ECAR) in young and aged female‐derived mammary tumor cell lines. (h) Western blot of ubiquitin lysine‐48 (Ub‐K48)‐linked proteins in young and aged female‐derived mammary tumors and quantification relative to Actin loading. (i) Quantification of the Western in h. (j) Proteasome activity in young and aged female‐derived mammary tumors. Each dot represents pooled technical (3) and biological replicates (7). (k) Proteasome activity in young and aged female‐derived mammary tumor cell lines. Each dot represents one technical replicate. (l) Western blot of XBP‐1s and MCL‐1 in lysates from young and aged female‐derived mammary tumors. (m) Quantification of XBP‐1s and MCL‐1 Western in L. (n) Percentage viability of young and aged female‐derived mammary tumor cell lines to the indicated dose of 4‐hydroxytamoxifen treatment for 48 h. (o) Western blot of XBP‐1 and CHOP in mammary glands of wild‐type mice with and without treatment with doxycycline as in Figure 1a. (p) Quantification of XBP‐1s and CHOP Western in O. (q) Western blot of SIRT3 in young and aged female‐derived mammary tumors. (r) Quantification of Western in Q. (s) On the left, plots of the distribution of the expression of XBP‐1 in patients aged 50 or less (267) or more than 50 (666). On the right, plots of the distribution of the expression of XBP‐1 in patients that are classified as ERα‐negative (179) or ‐positive (601). (t) Plot of the distribution of the expression of the 34 gene signature identified as being specific to aged female‐derived mammary tumors in Figure 1h in patients that are classified as ERα‐negative (179) or ‐positive (601)

We then focused on the 34 genes that are up‐regulated in the aged female‐derived tumors. Pathway analysis of these genes identified Nuclear Respiratory Factor‐1 (NRF‐1), a master regulator of the transcription of genes implicated in mitochondrial oxidative phosphorylation, the proteasome, and cytoplasmic translation (Figure 2a). We therefore aimed at validating these pathways. First, we performed a Western blot of NRF‐1 on mammary tumors derived from the young and aged mice and found that NRF‐1 is significantly up‐regulated in the aged mice (Figure 2b–c). To test whether the elevation in NRF‐1 results in alteration in respiration capacity, we first established cell lines from both young and aged female‐derived tumors. We confirmed that NRF‐1 is also elevated in the cell line derived from the aged mice (Figure S1a–b) and also tested for the level of ERα and PR in the cell lines. We found that unlike what is observed in the primary tumors in vivo, the expression of the ERα was much closer in the cell lines in vitro than in the primary tumors suggesting that expansion of cells in vitro imposes a selection for ERα‐positive cells (Figure S1c–d). However, the level of PR was drastically different between the young and aged female‐derived cells (Figure S1c–d). To confirm the expression of the ERα in the cell lines, we treated both cell lines with the ERα degrader fulvestrant. We found that treatment with fulvestrant eliminates the ERα band therefore confirming ERα expression in both cell lines (Figure S1e, f). We then performed Seahorse to monitor mitochondrial function. We found that both the basal and maximal respiration, which is based on measuring oxygen consumption rate (OCR), are significantly up‐regulated in the cell line derived from the aged mice (Figure 2d–e). Further, this observation is correlated with a decrease in extracellular acidification rate (ECAR), which measures the secretion of lactate as a by‐product of glycolysis (Figure 2f). Combined, the increase in respiration and decrease in ECAR resulted in a highly significant elevation in the baseline OCR/ECAR ratio in the aged female‐derived cells (Figure 2g). This finding indicates that aged female‐derived cancer cells rely mainly on oxidative phosphorylation for ATP generation, while the young female‐derived cells rely mainly on glycolysis.

To validate the proteasome, we performed a Western blot of ubiquitin lysine‐48 (Ub‐K48) linked proteins, which are marked for degradation by the proteasome. We found a significant decrease in the level of Ub‐K48 proteins in the aged female‐derived mammary tumors compared with the young female‐derived mammary tumors (Figure 2h–i). This decrease correlated with an increase in proteasome activity in both the mammary tumors (Figure 2j) and cell lines (Figure 2k).

Next, we aimed at understanding the increase in cytosolic translation detected by the RNAseq. (Figure 2a). One clue towards this goal is a recent study that revealed that the ERα is an RNA binding protein in the cytoplasm and can affect the splicing and translation of selected proteins involved in stress responses including splicing of XBP‐1 into XBP‐1s, which activates the XBP‐1 axis of the UPR of the endoplasmic reticulum (UPR^ER^) and translation of the anti‐apoptotic protein MCL‐1 (Xu et al., 2021). We therefore performed Western analysis of XBP‐1s and MCL‐1 in mammary tumors derived from the young and aged mice and found a significant increase in both XBP‐1s and MCL‐1 in the aged female‐derived samples (Figure 2l–m). Since upregulation of these stress pathways was reported to induce resistance to tamoxifen (Xu et al., 2021) and the association of XBP‐1 s expression and tamoxifen was also initially described by the Clark group (Clarke et al., 2011; Gomez et al., 2007), we also tested the relative sensitivity of the young and aged female‐derived cell lines to tamoxifen. In agreement with these previous reports, we found that the aged female‐derived cancer cells are more resistant to tamoxifen (Figure 2n). Since we used doxycycline to induce the expression of the oncogene in our mice and cell lines, and doxycycline was reported to induce the UPR^mt^ in *C. elegans* (Gao et al., 2022; Houtkooper et al., 2013), one concern was that doxycycline may impact the expression of the UPRs in the mammary gland. However, we previously reported that this is not observed for the UPR^mt^ in the mammary gland (Kenny, Craig, et al., 2019a); however, we had never examined the UPR^ER^ in this tissue after treatment with doxycycline. We therefore tested this possibility and found that doxycycline does not activate either XBP‐1 s or CHOP, two markers of the UPR^ER^ (Figure 2o–p). Collectively, these observations suggest that in the context of an aged microenvironment, luminal ERα‐positive cells may have a selective advantage over ERα‐negative cells based on their ability to activate cellular stress pathways that are regulated by the ERα.

Importantly, in addition to the XBP‐1 axis of the endoplasmic reticulum UPR, our group has reported that in breast cancer cell lines, mitochondrial stress activates an ERα axis of the mitochondrial UPR (UPR^mt^) (Papa & Germain, 2011). In this axis, the activation of the ERα is through phosphorylation by Akt, rather than estrogen, which is pertinent in the context of postmenopausal women. Activation of the ERα then leads to the up‐regulation of the master mitochondrial respiration transcription factor NRF‐1, which is a direct target of the ERα (Klinge, 2008, 2020; Mattingly et al., 2008). This ERα axis of the mitochondrial unfolded protein response (UPR^mt^) also leads to the up‐regulation of the activity of the proteasome (Papa & Germain, 2011), which acts as a guardian of the mitochondria by limiting the import of mutated or misfolded proteins in the mitochondria (Ng et al., 2021; Radke et al., 2008; Song et al., 2021).

Therefore, the pathways characterized by the 34 genes that are specifically up‐regulated in the aged female‐derived mammary tumors are consistent with the ability of the ERα to promote both the XBP‐1 axes of the endoplasmic reticulum UPR (UPR^ER^) and the ERα axis of the mitochondrial UPR (UPR^mt^), in addition to the up‐regulation of the anti‐apoptotic response through MCL‐1. These findings support the notion that the ERα is a major regulator of cellular stress responses and that this role of the ERα provides a selective advantage to ERα‐positive cells upon transformation in the physiological setting of aging.

In further agreement with this finding, we also reported that in ERα‐negative breast cancer cells, mitochondrial stress activates a distinct axis of the UPR^mt^ that is regulated by SIRT3 (Kenny et al., 2017; Kenny, Craig, et al., 2019a; Kenny, Gomez, & Germain, 2019b; Papa & Germain, 2014). Since SIRT3 decreases with age, it would argue that ERα‐negative cells may have a weaker ability to maintain mitochondrial function with age. This possibility was confirmed in a recent scRNAseq study of mammary cells over aging, where basal cells in aged mice were found to be characterized by a decrease in oxidative phosphorylation and electron transport chain capacity (Li et al., 2020). We tested the level of SIRT3 in the young and aged female‐derived mammary tumors and confirmed that SIRT3 is decreased in aged mice (Figure 2q‐r). This finding suggests that in absence of SIRT3, the maintenance of the integrity of the mitochondria may become more dependent on the ERα and contribute to the selection of luminal ERα‐positive cells in older women. The interdependence of the SIRT3 and ERα axes of the UPR^mt^ is further supported by the observation that SIRT3 knockout mice develop exclusively ERα‐positive mammary hyperplasia (Kim et al., 2010).

Importantly, a previous study reported the enrichment in the expression of luminal markers in CD49f^hi^ basal stem‐cell‐enriched population with aging (Dong et al., 2016) raising the possibility that the selection observed in our study could be due to the expression of the MMTV‐promoter in this subpopulation. However, this subpopulation of cells was not observed by scRNAseq in the aged mammary gland (Li et al., 2020). One major difference that may explain this discrepancy is the fact that in the Dong et al. study mice were between 26 and 32 months, while in the Li et al. study, aged mice are 14 months old, which is closer in age to the 18‐month‐old mice used in the current study. Collectively, however, these studies highlight the dynamic nature of the mammary gland during aging from young, aged, and elderly. In support of the highly dynamic nature of the mammary gland over aging, a very recent study demonstrated that the gene expression profiles and regenerative capacity of mammary stem cells are altered over the first 12 months of age and that gene signature related to oncogenesis are observed at later time points (Huang et al., 2022). These studies further support the need to expand the understanding of the link between aging and the mammary gland.

Finally, we aimed at validating the expression of XBP‐1 according to ERα status and age in human breast cancer. Interrogation of the TCGA database revealed increased XBP‐1 expression in both older patients (>50 years old) and in tumors with positive ERα status (Figure 2s). We then tested whether the entire 34 gene signature we identified in ERα‐positive mammary tumors in aged female mice also correlates with ERα status in humans and found that this signature is significantly associated with positive ERα status (Figure 2t). However, the association with age, while showing a trend, did not reach statistical significance. This is likely due to the multiple uncontrolled variables observed in humans that are not found in a controlled age‐matched mouse model.

In conclusion, the results presented in the current study offer a potential mechanism for the observation of the increased incidence of ERα‐positive breast cancer in older women. These results also indicate that, as in humans, aged mice may also have a bias towards luminal ERα‐positive mammary tumors and that age should be considered as an important modifier of mammary tumors subtype in future studies. Therefore, while the MMTV‐ErbB2 is considered an ERα‐negative model of mammary tumors, our data indicate that while this remains true in young mice when tumors in aged mice are analyzed, this model promotes the formation of ERα‐positive mammary tumors. Additionally, our data indicate that while the selection of cell lines in vitro favors the growth of ERα‐positive cells from both young and aged mice, age appears to affect the transcriptional programs mediated by the ERα since the downstream targets such as the PR and NRF‐1 are only observed in aged female‐derived ERα‐positive cells. Further, since these differences result in differential sensitivity to tamoxifen, this later observation suggests that future studies are required to understand the impact of age on the ERα transcriptional program to further enhance endocrine therapy.

## 2 EXPERIMENTAL PROCEDURES

### 2.1 Mice

All experiments were approved by the Mt. Sinai Institutional Animal Care and Use Committee (IACUC) and performed according to the principles of laboratory animal care outlined in NIH publication No. 86–23, revised 1985 edition. All experiments in mice were conducted in compliance with the ARRIVE guidelines. All mice were MMTV‐rtTA/TetO‐NeuNT (The Jackson Laboratory #010576) at either 3 months of age (young group) or 18 months of age (Aged group) (available from the Jackson Laboratory) at the time or sacrifice. All animals were maintained according to IACUC‐approved methods. The collected tissue was immediately frozen on dry ice and stored for later analysis.

### 2.2 Whole mounts

The fourth inguinal mammary glands were removed and partially air‐dried to standard microscope slides (Fisher Cat# 12–544‐3) for 5–7 min before fixing in 75% ETOH. 25% Glacial Acetic Acid overnight. Glands were then washed in 70% ethanol for 15 min before gradually changing to water. Carmine staining was performed in Carmine solution (Place 1 g carmine (Sigma C1022) and 2.5 g aluminum potassium sulfate (Sigma A7167) in 500 ml dH_2_O) overnight. Glands were then washed in MilliQ filtered water and dehydrated in an alcohol series. Finally, glands were cleared in xylene and mounted in Permount (Fisher Cat # SP15‐100).

### 2.3 Immunohistochemistry

Fresh tissue was fixed for at least 24 h in PBS buffered Formalin before embedding in paraffin and sectioning. Sections were then deparaffinized in xylene twice for 10 min each before being rehydrated through a decreasing ethanol series (100%, 90%, 70%) culminating in a gradual change to water. Antigen retrieval was performed in 10 mM Sodium citrate buffer pH 6 for 30 min at 90–100°C. Slides were then allowed to slow cool to room temperature and rinse twice in Tris‐buffered saline (TBS). Antibody staining was performed according to the manufacturer's suggested protocol for the ImmPRESS Excel Amplified Polymer Kit (Vectorlabs Cat# MP‐7601). ERα (Santa Cruz H184), Progesterone Receptor (Thermo Scientific MA1‐411) and Ki 67 (Abcam ab15580) were detected with sc7207 from Santa Cruz diluted 1:100 for 1 h at room temperature in a humidified chamber.

### 2.4 RNAseq

RNA sequencing was performed by the Mount Sinai Genomics Core Facility. FASTQ files were aligned to the mouse genome and analyzed using the BioJupies (Torre et al., 2018) with the default settings. BioJupies implements the limma method to identify differentially expressed genes. Gene Ontology analysis was performed using the suite of analysis tools in Enrichr (Kuleshov et al., 2016) Direct links to the Enrichr results are provided below:

34 genes unique comparing Aged 444 to Young 481:

https://maayanlab.cloud/Enrichr/enrich?dataset=fc1c719108796c0ff2524962d1affaea

71 genes unique comparing Aged 444 to Young 481:

https://maayanlab.cloud/Enrichr/enrich?dataset=04bf73b8fef6df870356b3c76fe3f72f

### 2.5 Western analysis

Western blot analysis was performed as previously described (Jenkins et al., 2021). Membranes were probed with primary antibodies against ERα (Santa Cruz H184), Progesterone Receptor (Thermo Scientific MA1‐411), K48Ub (EMD‐Millilore: cat. No. 05–1307), Actin (Santa Cruz Biotechnology: L cat. No. sc‐47,778, NRF‐1 (Abcam, cat. No. ab55744), MCL‐1 (Santa Cruz Biotechnology: cat. No. sc‐20,679), XBP‐1 (Santa Cruz Biotechnology: cat. No. sc‐7160), CHOP (Cell Signaling cat No. 2895S) or SIRT3 (Abcam: cat. No. ab264041).

### 2.6 Seahorse

Mitochondrial respiratory function was determined by a Seahorse XF24 extracellular flux analyzer (Seahorse Biosciences) as previously described (Chattopadhyay et al., In press). 25,000 cells were plated in each well of Seahorse plates in DMEM/F‐12 containing 10% FBS and 1% P/S overnight. 1 h prior to measurement, DMEM was replaced with Agilent Seahorse XF DMEM medium containing 1 mM pyruvate, 2 mM glutamine, and 10 mM glucose, pH 7.4. The assay was performed using Seahorse XF Cell Mito Stress Test Kit. The final concentrations of inhibitors used were 1 μM oligomycin, 1 μM FCCP (used as an uncoupler), and 0.5 μM complex III inhibitor antimycin A. Each plate (along with the cartridge) was loaded into the XF analyzer, and the OCR was measured under basal conditions and after the subsequent addition of oligomycin, FCCP, and rotenone.

### 2.7 Proteasome assay

The proteasome activity assay was performed as previously described (Jenkins et al., 2021).

### 2.8 Doxycycline treatment

MMTV‐rtTA/TetO‐NeuNT mice were induced with Doxycycline Hyclate (Sigma D9891) (1.5 g/L) in drinking water ad libitum for 6 weeks before sacrificing. Primary cell lines derived from these tumors were maintained in DMEM F‐12 plus 0.3 μg/ml doxycycline and 10% Fetal Calf Serum (FCS).

### 2.9 Cell viability assay and 4‐hydroxytamoxifen treatment

5000 cells per well were seeded in a 96‐well plate. Cells were cultured in phenol red‐free DMEM (Gibco 21: 063–029) plus 10% charcoal‐stripped FCS and were exposed to 4‐hydroxytamoxifen (Sigma H6278) in growth media with 1% FCS for 48 h before viability was assessed using the Crystal violet Assay Kit (Cell viability) from Abcam (ab232855) according to the manufacturer's instructions.

### 2.10 Collagen hybridizing peptide (CHP) staining

Biotinylated Collagen Hybridizing peptide (bCHP‐ 3Helix) was diluted in water at 20 μM. FFPE sections were deparaffinized and blocked with endogenous enzyme block (Dako S2003) for 30 min. Sections were then stained with 20 μM CHP overnight in a humidified chamber. Before staining, bCHP was heated at 85°C for 15 min and rapidly cooled on ice for 30 s with no more than 1–3 downtime minutes before being added to the section according to the manufacturer's recommendation. Sections were then washed in TBS and incubated with streptavidin‐conjugated HRP (1:150) (Vector Laboratories: SA5004) in antibody diluent (MP Biomedicals: 980,641) for 1 h at room temperature. After washing for 5 min in TBS, sections were then stained with DAB substrate (Thermo 34,002) for 30 min at room temperature. Sections were then quenched in water and counterstained in hematoxylin, and finally dehydrated and mounted in permount.

### 2.11 Decellularization

Freshly dissected mammary glands were decellularized in 1% SDS in TBS plus pen./step and DNase (1 U/ml) in 25 ml inside a 50 ml Falcon tube shaking at room temperature for 48 h with 6 full volume changes, followed by 48 hwith 6 full volume changes of water before being visualized by second harmonic generation (SHG). SHG images were then analyzed using OrientationJ in ImageJ (Puspoki et al., 2016; Rezakhaniha et al., 2012).

### 2.12 Masson trichrome stain

Formalin‐fixed paraffin‐embedded (FFPE) sections were stained using the Masson Trichrome Stain Kit (Epredia 87,019) according to the manufacturer's instructions. RGB color images of MTC stained FFPE sections were captured using a Zeiss Axiocam ERc 5 s. The color images were split into red green and blue channels using ImageJ. Image J was used to select and measure the areas positive for blue collagen staining.

### 2.13 Quantification of cytoplasmic ERα staining

Cells positive for ERα staining were manually counted and scored as either nuclear or cytoplasmic. The number of cells that were positive for cytoplasmic staining was then divided by the total number of cells in the field to determine the percentage of cells with cytoplasmic ERα staining. 5–10 fields were scored per animal.

### 2.14 TCGA human dataset analyses

Analysis of XBP‐1 and 34 genes signature in human breast cancer TCGA dataset (BRCA).

RNAseq normalized counts from the BRCA dataset were obtained from the UCSC Xena Hub: (https://tcga.xenahubs.net/download/TCGA.BRCA.sampleMap/HiSeqV2.gz). Clinical data were downloaded from the same resource at: (https://tcga.xenahubs.net/download/TCGA.BRCA.sampleMap/BRCA_clinicalMatrix.gz). A total of 933 specimens annotated with age were retained for this study. The enrichment score of the 34 genes signature specific to the aged female‐derived mammary tumors was calculated for each patient using a single‐sample gene set enrichment analysis (ssGSEA) from Gene Pattern 2.0 (ADD REF https://doi.org/10.1038/ng0506‐500). The gene expression of XBP‐1 or the enrichment score calculated as mentioned above for the 34 gene signature were correlated with age (equal or above 50 years old vs. below 50) and ER status (positive or negative). Statistical differences were established by the two‐sided Mann–Whitney Wilcoxon test.

## AUTHOR CONTRIBUTIONS

E.J.J has generated the data presented in Figure 1b,c,d,e,f,g,h,i,j,k,l,m and Figure 2a,b,c,e,f,g,h,i,j,k,l,m,n,o,p,q,r,s,t. M.C has performed the Seahorse analysis shown in Figure 2b,c,d,e,f,g. M.G with the help of N.S has generated the 3‐ and 18‐month‐old mice and induced tumor formation by doxycycline. A.M. and D.T. contributed to the initial analysis of the RNAseq data. M.G. generated the young and aged female‐derived mammary tumor cell lines.

## CONFLICT OF INTEREST

The authors have no conflicts of interest to declare.

## PATIENT CONSENT STATEMENT:

N/A.

## PERMISSION TO REPRODUCE MATERIAL FROM OTHER SOURCES

N/A.

## CLINICAL TRIAL REGISTRATION

N/A.

## Supporting information


Figure S1

Table S1

## Data Availability

All data is available upon request.
