# Supplementary figures and images for: Age alters the oncogenic trajectory toward luminal mammary tumors that activate unfolded proteins responses
Source: Aging Cell. 2022 Sep 15;21(10):e13665. doi: 10.1111/acel.13665 (PMC9577951; doi:10.1111/acel.13665)

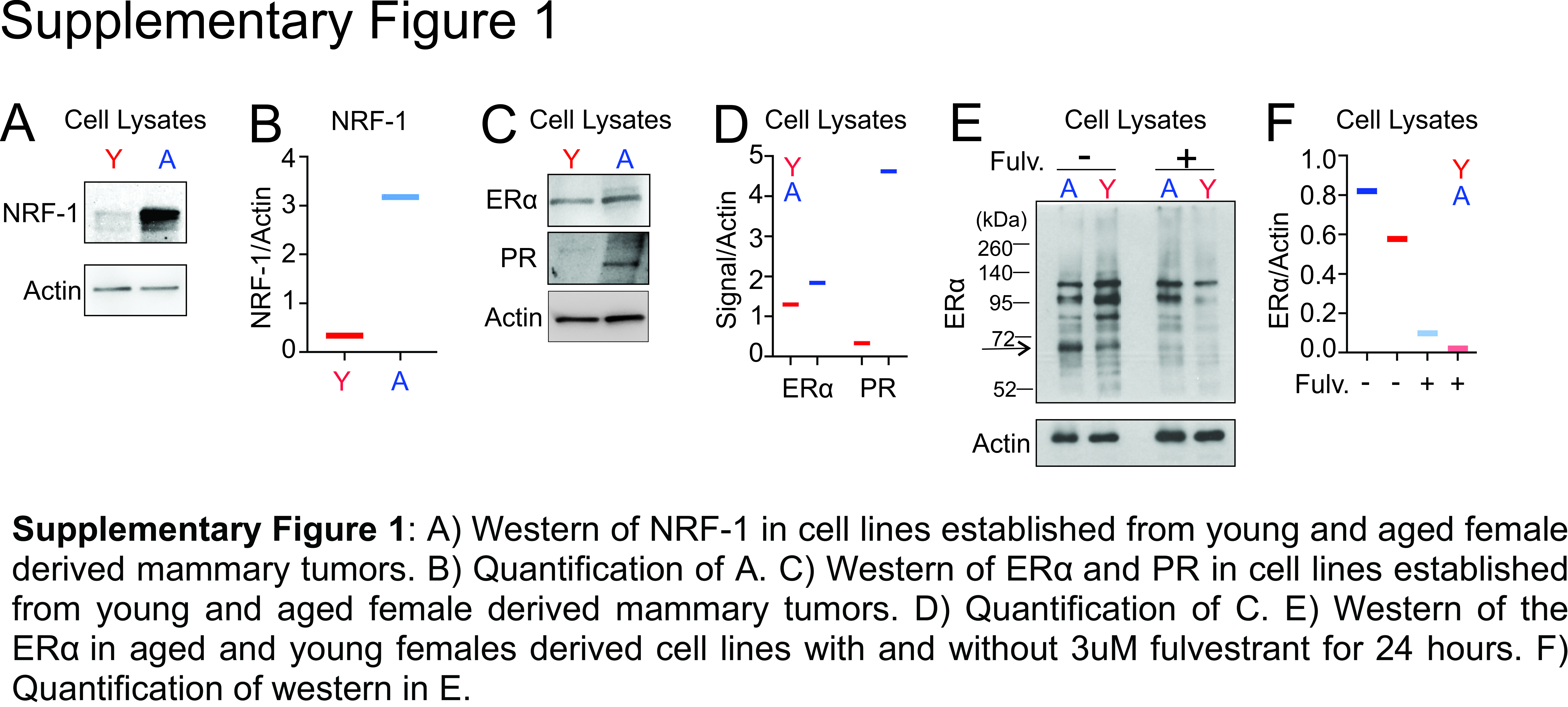

Supplement: Supplementary file 1 — Figure S1 [file ACEL-21-e13665-s001.tif]
