# Supplementary material for: Age alters the oncogenic trajectory toward luminal mammary tumors that activate unfolded proteins responses
Source: Aging Cell. 2022 Sep 15;21(10):e13665. doi: 10.1111/acel.13665 (PMC9577951; doi:10.1111/acel.13665)
Supplement: Supplementary file 2 — Table S1 [file ACEL-21-e13665-s002.docx]

Supplementary Table 1:

**71 genes up-regulated in young-females derived mammary tumors**:

Cd74

Tuba1a

Igfbp7

H2-Ab1

Amfr

Papss1

Scd1

Tagln2

Slpi

Col4a2

Muc15

Trps1

Smox

Mdh2

Fkbp8

Erp29

Plekhb1

Nedd8

Pla2g7

Vim

Cldn3

Cpe

Rbp1

Ifitm3

Cst3

Col9a1

Myh9

Ybx1

Hist1h4j

Rpn1

Gabrp

Ltf

Wdr1

Csn2

Cldn7

Emb

Cd151

Ier3

Emp1

Csn1s1

Vdac1

Serpine2

Col3a1

Aldoc

F11r

Aqp5

Krt19

Aplp2

Ppap2c

Cttn

Tax1bp1

Igf2r

Krt14

Srsf5

Igfbp2

Psmd4

Plet1

S100a16

Cnn3

Pfkp

Tnc

Clca3a2

Stat3

Sparc

Cltc

Ano1

Car2

Mlec

Col4a1

Csn1s2a

**34 genes up-regulated in aged-females derived mammary tumors:**

Tram1

Snrpe

Hist1h4c

Arl1

Ndufa6

Gm10052

Capza2

Eif3c

Eif3m

Eef1d

Ppp2ca

Psmb6

Prdx5

Ran

Rnaset2a

Paics

Uqcrb

Myl12b

Sh3bgrl

Eif4a2

Tspo

Lamp2

Cyba

Psmb3

Atp6v0e

Vdac2

Ptp4a2

Rabac1

Swi5

Eif3e

M6pr

Gng5

Crip1

Eif3k
